# Exploring the Use of Artificial Intelligence Techniques to Detect the Presence of Coronavirus Covid-19 Through Speech and Voice Analysis

**DOI:** 10.1109/ACCESS.2021.3075571

**Published:** 2021-04-26

**Authors:** Laura Verde, Giuseppe De Pietro, Ahmed Ghoneim, Mubarak Alrashoud, Khaled N. Al-Mutib, Giovanna Sannino

**Affiliations:** Institute of High—Performance Computing and Networking (ICAR)-National Research Council of Italy (CNR) 80131 Naples Italy; Department of Software EngineeringCollege of Computer and Information SciencesKing Saud University 37850 Riyadh 11543 Saudi Arabia; Mathematics and Computer Science DepartmentFaculty of ScienceMenoufia University 68849 Shebin El-Koom 32511 Egypt

**Keywords:** Artificial intelligence techniques, Covid-19~detection, speech analysis, voice analysis

## Abstract

The Covid-19 pandemic represents one of the greatest global health emergencies of the last few decades with indelible consequences for all societies throughout the world. The cost in terms of human lives lost is devastating on account of the high contagiousness and mortality rate of the virus. Millions of people have been infected, frequently requiring continuous assistance and monitoring. Smart healthcare technologies and Artificial Intelligence algorithms constitute promising solutions useful not only for the monitoring of patient care but also in order to support the early diagnosis, prevention and evaluation of Covid-19 in a faster and more accurate way. On the other hand, the necessity to realise reliable and precise smart healthcare solutions, able to acquire and process voice signals by means of appropriate Internet of Things devices in real-time, requires the identification of algorithms able to discriminate accurately between pathological and healthy subjects. In this paper, we explore and compare the performance of the main machine learning techniques in terms of their ability to correctly detect Covid-19 disorders through voice analysis. Several studies report, in fact, significant effects of this virus on voice production due to the considerable impairment of the respiratory apparatus. Vocal folds oscillations that are more asynchronous, asymmetrical and restricted are observed during phonation in Covid-19 patients. Voice sounds selected by the Coswara database, an available crowd-sourced database, have been e analysed and processed to evaluate the capacity of the main ML techniques to distinguish between healthy and pathological voices. All the analyses have been evaluated in terms of accuracy, sensitivity, specificity, F1-score and Receiver Operating Characteristic area. These show the reliability of the Support Vector Machine algorithm to detect the Covid-19 infections, achieving an accuracy equal to about 97%.

## I. Introduction

The emergence of coronavirus has been considered a major threat to public health in almost all countries around the world during the last year. Millions of lives have been and are currently being disrupted by this pandemic. More than 80 million confirmed Covid-19 positive cases worldwide since the pandemic began have been recorded, and more than one million deaths, numbers which are, unfortunately, constantly increasing. [1].

Health services and network resources around the world have been put to a severe test [2]. Timely treatment for many patients is needed, as well as early diagnosis and monitoring, with healthcare workers aiming to utilize the limited resources available most effectively. On account of its high infection rate, the development of techniques able to identify the presence of Covid-19 and distinguish it from other forms of influenza and pneumonia, in a fast and reliable way, is crucial.

Internet of Things (IoT) and Artificial Intelligence (AI) can play a significant role by offering a better insight into healthcare data, and by supporting affordable personalized care, often, by using opportune wereable sensors [3], [4]. It is possible, not only, to improve the processing and storage facilities of huge IoT data streams (big data), but also to offer quality patient care through faster and more reliable diagnosis systems which make use of AI algorithms [5]–[9]. The progress of the IoT, cloud and edge computing, wireless communication, mobile health systems, and reliable AI algorithms have, in fact, contributed to an improvement in the diagnosis and treatment of various diseases. Several monitoring systems effective in managing chronic conditions and emergencies have been proposed in the last few years [10]–[17]. These systems offer different functionalities, such as for the collection and analysis of health data necessary for the real-time monitoring, and accurate and faster processing of patient data.

In this work, we investigate the possibility of supporting the early detection and assessment of the presence of the Covid-19 infection through the analysis of voice sounds using machine learning (ML) algorithms. The aim is to identify the most reliable ML technique in terms of the detection of voice alterations due to Covid-19 and embed this within a smart mobile healthcare solution for the accurate distinction between pathological and healthy subjects. Although, in fact, the World Health Organisation (WHO) currently recommends the diagnosis of Covid-19 using molecular tests in laboratories [18], the tracking of the virus globally and to diagnosis of the pathology at an early stage could considerably benefit from this solution. It would be ideal for an easy, portable, non-invasive and low-cost mass screening phase since the analysis of the sound of the voice can be acquired through a mobile device, such as a smartphone or tablet.

The hypothesis outlined in an interesting paper published in September 2020, in the IEEE Open Journal of Engineering in Medicine and Biology, was that “Covid-19 subjects, especially asymptomatics subjects, could be accurately discriminated from healthy individuals by using only a forced-cough cell phone recording realized by means Artificial Intelligence” [19]. Therefore, starting from this important hypothesis, our idea has been to find the best ML technique in terms of detecting the presence of Covid-19, especially in asymptomatic individuals, who are considered as “silent drivers” of the pandemic. In fact, although symptomatic people have been identified to be the primary source of SARS-CoV-2 transmission, there is a high possibility of transmission via asymptomatic individuals. Due to the absence of symptoms, such subjects are the most difficult to track.

Recent studies have shown anomalies in oscillation of the vocal folds during phonation in Covid-19 patients [20], [21], including asymptomatic subjects. These individuals not only report changes in their voice, but also a general inability to produce their voice normally. Therefore, in our study, sounds of the vowels /a/, /e/ and /o/ selected from the Coswara database, an available crow sourced database [22] were processed to extract appropriate features to be used as inputs of the main machine learning algorithms. The performances of these techniques were evaluated in terms of accuracy, F1-score, sensitivity, specificity and Receiver Operating Characteristic (ROC) area.

The remaining sections of the paper are organized as follows. Section II introduces the studies existing in literature concerning the detection of Covid-19 disorders about the voice quality using machine learning techniques. Section III, instead, presents the dataset, features and algorithms evaluated in this study, while the classification results are reported and discussed in Section IV. Finally, our conclusions are presented in Section V.

## II. Related Works

Several studies have proposed automatic Covid-19 screening by analysing chest radiographic [23]–[25] or Computed Tomography (CT) images [26]–[28], identifying the pathomorphological alterations caused by Covid-19 in the patients chest. Other authors, instead, have recommended the analysis of coughing [19], [29]–[32] or respiratory signals [33], [34], to detect the presence of alterations due to the Covid-19 infection.

A few other studies, instead, have indicated solutions for the detection of Covid-19 disorders based on an analysis of voice samples. Han *et al.* [35] proposed a system capable of estimating the severity of the illness by means of an assessment of five sentences collected from 52 Covid-19 patients in two hospitals in Wuhan, China. Two acoustic feature sets were considered, the Computational Paralinguistics Challenge set and the Geneva Minimalistic Acoustic Parameter set. A Support Vector Machine (SVM) algorithm classified the voice signals, achieving an accuracy equal to 69%.

Mel filter bank features constitute the alternative input to the SVM model, as proposed in [36]. Experiments were performed on a small dataset (10 pathological and 9 healthy subjects) collected from You Tube videos, obtaining an accuracy and F1 score, respectively, equal to 70.5% and 77.0%.

Spectral Centroid (SC), Spectral Roll-off (SR), Zero-Crossing rate (ZCR), Mel-Frequency Cepstral Coefficients (MFCC), the first and second derivates of MFCC are, instead, features extracted from the coughing, breathing and vocal sounds of a private database constituted by 80 subjects (20 pathological and 60 healthy) in [34]. These were processed with the Long Short-Term Memory (LSTM) architecture, achieving an,F1-score and accuracy, concerning to the voice samples., equal to 92.5% and 88.2%, respectively.

A Convolutional Neural Network (CNN) model that locates anomalies in the dynamics of the glottal flow waveform (GFW) during voice production was proposed in [37]. This is able to identify the features most significant for Covid-19 detection from 19 voice samples of the vowels /a/, /i/ and /u/. (10 healthy and 9 pathological) from a private database collected by a private company in Chile.. The performance of the model was evaluated in terms of the area under the curve of the Receiver Operating Characteristic (ROC-AUC) and its standard deviation, respectively equal to 0.900 and 0.062, in an analysis of the vowels /i/ and /u/.

In summary, there are very few studies in the literature relating to the detection of Covid-19 disorders through an analysis of vocal sounds, probably due to the recent dissemination of the virus and the continuing development of the pandemic. Most of these works have been performed on very limited and, often, non-accessible datasets, a fact which reduces their access to the wider research community and limits the further development of classification techniques on standardized datasets.

## III. Materials and Methods

In order to investigate the most reliable ML technique capable of detecting alterations due to the Covid-19 infection through an analysis of vocal sounds. Appropriate voice samples were selected from an available database, the Coswara database. For each subject, the voice sounds of three vowels, /a/, /e/ and /o/, were processed to extract opportune features used as the input to the algorithms analysed. Additionally, we have tested also a combination of these features extracted by the three vowels, and this combination has achieved better results in terms of correct classification between healthy and pathological subjects.

The following subsections report additional details about the voice dataset used to estimate the classification accuracies of the various ML models, as well as the features extracted from each voice sample and the techniques considered.

### A. Database

**FIGURE 1.**
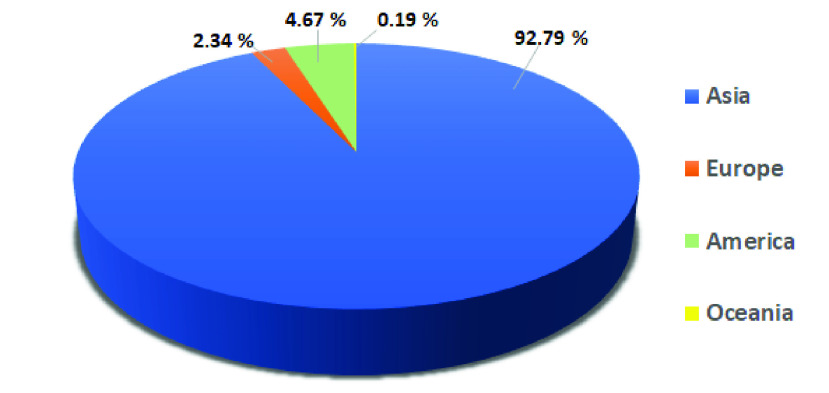
Geographical location of the subjects involved in this study.

Coswara is an available crowd-sourced database accessible on the GitHub platform [38], realised by the Indian Institute of Science (IISc) Bangalore. The aim of the project, named Coswara after a combination of the words Covid-19 and Swara (sound in Sanskrit), is to collect sound samples to provide a database useful for an evaluation of the reliability of technologies to support the diagnosis of Covid-19 [22]. Coughing, breathing and voice sounds were collected from each subject, in addition to data relating to health status, gender, age, certain pre-existing health conditions (i.e. asthma, diabetes) and geographical location. The samples were recorded in all continents, except Africa, with a prevalence of sounds coming from Asia, as shown in Figure 1.

In this preliminary study, only voice alterations due to the Covid-19 infection were estimated, the sustained phonation of the vowels /a/, /e/ and /o/ was evaluated. The use of the vowel sound is particularly effective for an evaluation of the voice quality because it avoids any linguistic artifacts due to the different languages of the subjects involved in the project [39]. The choice to evaluate the effects of Covid-19 infections by using only the voice sounds is made because we would like to realise a smart mobile healthcare solution able to detect and assess the presence of Covid-19 infections through the simple analysis of voice sounds. It would be ideal for an easy, portable, non-invasive and low-cost mass screening phase since the analysis of the sound of the voice can be acquired through a mobile device, such as a smartphone or tablet.

All the recordings were sampled at 44.1 KHz and their resolution is 32-bit. An opportune filter was applied to reduce the noise added during the acquisition [40]. Voice sounds particularly corrupted by noise and of a duration of less than one second were excluded. In detail, our dataset was composed of voice samples from 1,027 subjects, 77 Covid-19 positive (54 male and 23 female) and 950 healthy (721 male and 229 female). The low number of female samples could be related to the higher incidence of Covid-19 disorders in male subjects than in females [41], [42].

Table 1 shows further details about the samples used in this study, in which we have reported the number (No) of the voices considered for each gender, and each health status. Additionally, it also shows the percentage of each item calculated in relation to each category.

**TABLE 1:** Details of the Subjects Involved in this Study

Category	Age Group	Female subjects		Male subjects	
		No.	%	No.	%
Healthy	≤25	85	8.9%	220	23.2%
	26–45	111	11.7%	382	40.2%
	46–65	29	3.1%	111	11.7%
	$\geqslant 66$	4	0.4%	8	0.8%
Total		229	24,1%	721	75.9%
Covid-19 positive	≤25	13	16.9%	27	35.1%
	26–45	4	5.2%	19	24.7%
	46–65	6	7.8%	8	10.4%
	$\geqslant 66$	0	0%	0	0%
Total		23	29.9%	54	70.1%
All	≤25	98	9.5%	247	24.1%
	26–45	115	11.2%	401	39.0%
	46–65	35	3.4%	119	11.6%
	$\geqslant 66$	4	0.4%	8	0.8%
Total		252	24.5%	775	75.5%

### B. Feature Extraction

The choice of the appropriate features that can represent the data in a relevant way is fundamental in that this can have a significant impact on the reliability of the system. Therefore, we evaluated not only the main parameters used in clinical practice to assess voice quality, such as Fundamental Frequency (F_0_), jitter, shimmer and Harmonic to Noise Ratio (HNR) [43] but also other well-known parameters used in literature for the voice classification, such as Mel-Frequency Cepstral Coefficients (MFCC) or Spectral Centroid or Roll-Off [44]–[47]. This choice has been made due to the fact that the diffusion of the Covid-19 pandemic is very recent, so, thus far, there isn’t any specific medical protocol for the assessment of Covid-19 disorder by analysing voice sounds, and no sufficient studies are present in the literature concerning the evaluation of which features are more reliable in determining vocal changes due to the Covid-19.

In detail, the following features were evaluated:

• Fundamental Frequency (F_0_): this represents the rate of vibration of the vocal folds, used to evaluate the correct functioning of the larynx.
• Jitter and Shimmer: these features indicate the instability of the oscillating pattern of the vocal folds used to evaluate the cycle-to-cycle changes in frequency (jitter) and amplitude (shimmer).
• Harmonic to Noise Ratio (HNR): this shows the presence of noise in the voice signal, due to an incorrect vocal fold closure resulting from the onset of a disorder.
• Mel-Frequency Cepstral Coefficients (MFCC): these coefficients constitute a representation of the voice signal, based on the linear cosine transform of a log power spectrum on a non-linear mel scale of frequency. We used the first 13 components.
• First and second derivatives of cepstral coefficient: these evaluate the dynamic behavior of the voice signal.
• Spectral Centroid (SC): this represents the center of the mass of the spectrum, useful for an evaluation of the changes of the signal frequency over time.
• Spectral Roll-off (SR): this indicates the slope of the voice signal spectrum. It is used to distinguish voiced and unvoiced sounds.

F_0_, jitter, shimmer and HNR were estimated using the Java Programming Language through the use of Eclipse IDE (version 4.6.3) according to the procedures indicated in [48]–[50]. The MFCC, derivatives of the cepstral coefficient, SC and SR were, instead, calculated by using Matlab, version R2020a, adopting the function *audioFeatureExtractor* [51].

### C. Machine Learning Techniques

Several machine learning algorithms were evaluated in order to identify the most reliable technique correctly distinguish between a healthy and pathological voice. All the experiments were performed adopting one of the most popular frameworks used for classification in machine learning, the Waikato Environment for Knowledge Analysis project (WEKA) [52], version 3.8.4, selected on account of its affordability, efficiency and versatility in the analysis of data. We used a machine with 8 GB memory and Intel(R) Core(TM) i5-6200U CPU with 2.40 GHz.

The ML techniques are divided into several groups:

• **Bayes**: algorithms that use the Bayes theorem to classify the data. The probabilistic model, used in the classification task, is represented by a set of random variables and their conditional dependencies. In this study, the *Naive Bayes (NB)* and *BayesNet (BN)* algorithms were used [53].
• **Functions**: this category includes an assorted group of classifiers whose operation can be described as a mathematical equation. The performance of the *Support Vector Machine (SVM)* [54] and *Stochastic Gradient Descent (SGD)* [55] were evaluated in this study.
• **Lazy**: specific instances are used by these approaches to classify the data. The *k-nearest neighbor (Ibk)* [56] and *Locally weighted learning (LWL)* [57] classifiers were analysed in this study.
• **Meta**: these techniques combine the multiple machine learning models to improve the classification accuracy with a consequent increase of network complexity and computational time [58]. The *Adaboost* and *Bagging* algorithms were evaluated in this study.
• **Rules**: these algorithms use rules to classify the data. *One-R* [59] and *Decision Table (DT)* [60] are two algorithms belonging to this category.
• **Trees**: this approach represents the learned function as a decision tree. Generally, it is used to classify categorical data. In this study, we evaluated the performance of the *C4.5 decision tree (J48)* [61] and *REPTree* algorithms.

For each category, the performances of the best algorithms are reported in this study. Additionally, in this preliminary experimental phase, the setting parameters for each classifier are the same as Weka’s default values.

## IV. Results and Discussion

The classification reliability of the selected machine learning techniques was evaluated by dividing randomly the voice samples of the dataset into training and testing sets. 80% of the samples constituted the training set, while the remaining 20% constituted the testing set. In detail, voice samples (the vowels /a/, /e/ and /o/) of 822 subjects (62 pathological and 760 healthy) were selected for the training set, while recordings (the vowels /a/, /e/ and /o/) of 205 subjects (15 pathological and 190 healthy) compose the test set.

The performances of the machine learning algorithms were evaluated in terms of accuracy, specificity, F1-score, recall, precision and Receiver Operating Characteristic (ROC) area. These metrics were calculated by defining as True Negatives (TN) or True Positives (TP) the number of cases correctly classified, respectively, as healthy or pathological. False Negatives (FN) or False Positives (FP) represent, instead, the number of cases incorrectly classified, respectively, as healthy or pathological.

Accuracy indicates the number of correct predictions over all the input samples. It is expressed by the equation 1:

\begin{equation*} Accuracy =\frac {TP+TN}{TP+TN+FP+FN}\tag{1}\end{equation*}

Specificity, instead, measures how many healthy predictions made are correct and is calculated according to the equation 2:

\begin{equation*} Specificity =\frac {TN}{TN+FP}\tag{2}\end{equation*}

Since dataset have uneven class distributions, F1-score can well illustrate the behavior of a model when the data sets are disproportionate. This provides a measure of a model’s accuracy and is, generally, described as the harmonic mean of the recall and precision, that is:

\begin{equation*} F1-score = 2*\frac {precision*sensitivity}{precision+sensitivity}\tag{3}\end{equation*}

where precision is a measure of how many of the positive predictions made are correct (true positives), while the sensitivity measures how many of the positive cases the classifier correctly predicted, over all the positive cases in the dataset. These were calculated, respectively, as:

\begin{align*} Precision=&\frac {TP}{TP+FP} \tag{4}\\ Sensitivity=&\frac {TP}{TP+FN}\tag{5}\end{align*}

Finally, the area under the ROC curve (AUC) was estimated. This evaluates the goodness of the classifier, when the AUC is minimum (AUC = 0), the algorithm incorrectly classifying all voice samples, and when the AUC is maximum (AUC = 1), the model distinguishing perfectly between the healthy and pathological voice samples.

Tables 2 and 3 show the results achieved, respectively, on the training and testing sets. SVM and SGD obtained the best classification results, for the training and testing experiments. In detail, SVM distinguishes healthy and pathological subjects with an accuracy equal to about 97%, and specificity and recall equal, respectively, to approximately 97% and 93%. This means that this approach classifies correctly both healthy and pathological subjects. It is confirmed by the F1-score result (about 82%), which represents one of the most reliable metrics to measure the goodness of the algorithm due to the high imbalance between healthy and pathological samples in the dataset considered. The lowest performances are, instead, achieved by *Rules* algorithms. These obtained low precision and recall results, being more affected than SVM by the low number of Covid-19 positive cases compared to healthy ones in the dataset considered.

**TABLE 2:** Results Achieved on the Training Set

Category	Algorithm	Accuracy (%)	F1-score (%)	Specificity (%)	Precision (%)	Recall (%)	AUC
Bayes	NB	95.13	75.31	94.87	61.00	98.39	0.949
	BN	93.31	69.27	92.76	52.99	100	0.964
Functions	SVM	100	100	100	100	100	1.000
	SGD	100	100	100	100	100	1.000
Lazy	Ibk	100	100	100	100	100	1.000
	LWL	93.80	70.86	93.29	54.87	100	0.999
Meta	Adaboost	98.18	89.21	98.03	80.52	100	0.998
	Bagging	98.66	91.20	99.21	90.48	91.94	0.998
Rules	OneR	96.47	74.78	98.68	81.13	69.35	0.840
	DT	97.93	85.95	99.08	88.14	83.87	0.981
Tree	J48	99.39	95.93	99.74	96.72	95.16	0.998
	REPTree	95.99	74.81	97.37	71.01	79.03	0.972

**TABLE 3:** Results Achieved on the Testing Set

Category	Algorithm	Accuracy (%)	F1-score (%)	Specificity (%)	Precision (%)	Recall (%)	AUC
Bayes	NB	90.24	54.55	91.05	41.38	80.00	0.861
	BN	89.76	58.82	88.95	41.67	100	0.945
Functions	SVM	97.07	82.35	97.37	73.68	93.33	0.954
	SGD	95.12	66.67	97.37	66.67	66.67	0.820
Lazy	Ibk	93.17	50.00	96.84	53.85	46.67	0.718
	LWL	89.76	58.82	88.95	41.67	100	0.971
Meta	Adaboost	91.22	55.00	92.63	44.00	73.33	0.963
	Bagging	92.20	50.00	95.26	47.06	53.33	0.965
Rules	OneR	88.78	34.29	92.63	30.00	40.00	0.663
	DT	91.22	35.71	95.79	38.46	33.33	0.829
Tree	J48	93.66	60.61	95.79	55.56	66.67	0.954
	REPTree	91.22	52.63	93.16	43.48	66.67	0.948

Due to the short time frame, the continuing development of the pandemic and the difficulty in collecting data, the dataset is relatively imbalanced, the number of pathological voices being lower than healthy ones. To avoid the effect of this limitation, the F1-score values for each technique were calculated. Meanwhile, data collection is still in progress. Additional data will allow a more in-depth analysis, so improving the performance of the model, evaluating not only machine learning algorithms but also deep learning ones, and rendering it more robust and reliable. In the next evaluations, it will be possible to increase the numerosity of samples, adopting appropriate augmentation techniques. However, in the future, it will be necessary not only to increase the number of the collected samples but also to improve the quality of these samples. Currently, all the available databases are crowd-sourced, with all the samples independently recorded by volunteers. To validate an approach useful for the early detection of Covid-19, a controlled clinical trial is needed, since it is essential to have items labeled by medical experts. Moreover, due to the rapid and very recent diffusion of this pandemic, the information about the causes and developments of this disease, as well as the relationship with demographic and clinical data of patients suffering from Covid-19, is still few. In this preliminary study, we exclusively evaluated the effects of Covid-19 infection on voice quality. Nevertheless, as future plans, we want to analyse also the effects of patient’s data, such as age and gender, the etiopathogenesis of the pandemic, the symptoms of which, especially in the early stages of the disease, are still very often confused with other respiratory infections, to detect Covid-19 disorders and make possible improvements to the reliability of the model.

## V. Conclusion

The rapid spread of Covid-19 and the high infection and mortality rates have put a strain on global health systems. The delay in developing an effective vaccine and therapy against Covid-19 has made it necessary to implement unprecedented virus containment and mitigation measures to avoid public health collapse and minimize the risk of transmission, including not only communication campaigns, the sanitization of environments and the immediate isolation of symptomatic cases, but also social distancing and restrictions on population movement.

The contribution that IoT technologies and the analysis of Big data through AI algorithms can make in terms of containing the spread of the virus is fundamental. The utility of these measures in providing care to the population by limiting physical contact as well as achieving an early detection and monitoring of the health of Covid-19 patients in a faster, more reliable and cheaper way is indisputable.

In this paper an investigation into the classification accuracy of the main ML techniques in the detection of the presence of Covid-19 through voice analysis has been proposed. The aim has been to identify the most reliable technique and to embed it within a mobile health solution, In these terms, our objective has been to realize a system capable of supporting the early detection of the Covid-19 disorder, which could be useful as a fast pre-screening test as well as for the monitoring of patients’ symptoms, so reducing the burden on national health services.

Several ML techniques have been applied in an analysis of voice samples selected from a crowd-sourced database, the Coswara database, freely available. The results have shown that the best accuracy in Covid-19 detection is achieved by the SVM technique. This algorithm distinguishes a pathological and healthy voice with an accuracy equal to about 97%.
